# Role of Retinal Amyloid-β in Neurodegenerative Diseases: Overlapping Mechanisms and Emerging Clinical Applications

**DOI:** 10.3390/ijms22052360

**Published:** 2021-02-26

**Authors:** Liang Wang, Xiaobo Mao

**Affiliations:** 1 Miller School of Medicine, University of Miami, Miami, FL 33136, USA; liang.wang@med.miami.edu; 2 Neuroregeneration and Stem Cell Programs, Institute for Cell Engineering, Johns Hopkins University School of Medicine, Baltimore, MD 21205, USA; 3 Department of Neurology, Johns Hopkins University School of Medicine, Baltimore, MD 21205, USA

**Keywords:** amyloid-β, Alzheimer’s disease, glaucoma, age-related macular degeneration

## Abstract

Amyloid-β (Aβ) accumulations have been identified in the retina for neurodegeneration-associated disorders like Alzheimer’s disease (AD), glaucoma, and age-related macular degeneration (AMD). Elevated retinal Aβ levels were associated with progressive retinal neurodegeneration, elevated cerebral Aβ accumulation, and increased disease severity with a decline in cognition and vision. Retinal Aβ accumulation and its pathological effects were demonstrated to occur prior to irreversible neurodegeneration, which highlights its potential in early disease detection and intervention. Using the retina as a model of the brain, recent studies have focused on characterizing retinal Aβ to determine its applicability for population-based screening of AD, which warrants a further understanding of how Aβ manifests between these disorders. While current treatments directly targeting Aβ accumulations have had limited results, continued exploration of Aβ-associated pathological pathways may yield new therapeutic targets for preserving cognition and vision. Here, we provide a review on the role of retinal Aβ manifestations in these distinct neurodegeneration-associated disorders. We also discuss the recent applications of retinal Aβ for AD screening and current clinical trial outcomes for Aβ-associated treatment approaches. Lastly, we explore potential future therapeutic targets based on overlapping mechanisms of pathophysiology in AD, glaucoma, and AMD.

## 1. Introduction

Accumulation of amyloid-β (Aβ) in the retinal layers has been implicated as a key overlapping feature between three neurodegeneration-associated disorders that have affected millions of older adults worldwide: Alzheimer’s disease (AD), glaucoma, and age-related macular degeneration (AMD). All three disorders are chronic, age-related disorders with no known cure and can lead to irreversible disability [1,2,3]. In AD, accumulation of Aβ in the central nervous system (CNS) has been suggested to induce neurodegeneration especially in the hippocampus leading to progressive loss of cognitive function [4]. Visual disturbances and retinal Aβ accumulations have been reported in patients with early or even preclinical AD with retinal Aβ deposits appearing to be detected earlier than neurodegeneration and associated cerebral Aβ in AD mice models [5,6]. Similarly, glaucoma is characterized by retinal neurodegeneration most commonly in relation to elevated intraocular pressure (IOP). In glaucoma animal models, Aβ has been identified to be associated with increased retinal ganglion cell (RGC) susceptibility to elevated IOP and purposed to induce RGC apoptosis and optic nerve (ON) degeneration [7]. For AMD, severe central vision loss occurs after disruption of the retinal pigmental epithelium (RPE) with the formation of drusen, which leads to retinal neuronal degeneration, especially in the photoreceptor cells (PRC) [8]. Through postmortem studies and AMD mice models, Aβ deposits have been identified inside RPE cells and drusen that has been suggested to be associated with AMD progression [8,9,10].

Epidemiological connections have also been determined between AD, glaucoma, and AMD. Both glaucoma and AMD also appear to be related to a decline in cognitive function, although it is unclear if subjects had other undetected co-existing underlying pathologies [11,12,13,14]. Patients who have glaucoma and AMD have been associated with an increased risk for AD [15]. Similarly, AD patients have an increased prevalence for glaucoma with glaucoma observed in 7–24% of AD patients in comparison to 4–10% of healthy controls [16]. Advanced AMD prevalence was also doubled in AD patients in comparison to controls. However, this association was not obvious after correcting for shared risk factors such as age, presence of an apolipoprotein E allele, and smoking [17]. Since Aβ accumulation in the retina is considered to be a mechanistic link between these degenerative diseases, this warrants further exploration and cross-examination of the pathophysiological role of retinal Aβ and its implications for disease monitoring and treatment. We describe recent understandings of how retinal Aβ presents in AD, glaucoma, and AMD. Then, we evaluate existing findings of clinical trials and discuss potential retinal Aβ-associated mechanisms that may provide novel targets for therapeutic interventions.

## 2. Aβ in the Retina

As a developmental outgrowth of the diencephalon, the retina is the innermost layer of the eye that shares structural and pathophysiological pathways with the CNS including a connection between the microvasculature and axonal projections [18], and contain a diverse population of neurons [19,20]. The exact role of Aβ in the eye is still unknown, although Aβ has been suggested to have an anti-microbial effect in the brain, which may also apply to its role in the retina [21]. Aβ is a 39–43 amino acid protein peptide that originates from the amyloidogenic pathway with cleavage of a transmembrane glycoprotein, amyloid precursor protein (APP), by β- and γ-secretase [22,23]. APP is expressed in various tissues including the retina and appears to support synaptogenesis and neuronal development and survival [23]. Through the non-amyloidogenic pathway, proteolytic processing of APP by α- and γ-secretase generates soluble amyloid precursor protein (sAPPα), which has been shown to have a neuroprotective function in the retina [24]. Within the retina during pathological states, these Aβ monomers have been observed to spontaneously aggregate into dimers, trimers, and oligomers [22]. Through hyperspectral Raman imaging, soluble Aβ oligomers have been demonstrated to self-assemble into beta-pleated sheets and form structures such as protofibrils, fibrils, and insoluble amyloid plaques through hydrogen bonding between peptide bonds of parallel oligomers [22,25]. Similar to Aβ in the brain, in murine models [26], retinal Aβ oligomers have been shown to be more neurotoxic than fibrils with smaller oligomers formations associated with increased amounts of neuronal loss. Fibrillar structures have also been observed to shift to oligomer structures in vivo [23]. The main alloforms of Aβ in the retina are Aβ42 and Aβ40 [22]. Aβ42 has been observed to be more neurotoxic in the retina even though Aβ40 has been more commonly found throughout the retina for these neurodegeneration-associated disorders. The exact Aβ42/Aβ40 ratios in the retina will need to be determined in future studies [23,27].

## 3. Alzheimer’s Disease

Around 50 million older adults have been diagnosed with dementia worldwide and AD is the most common cause of dementia [28]. Aβ deposits that form insoluble plaques in the brain regions have been purposed to be the principal source of neurotoxicity that leads to brain atrophy and cognitive decline. The highest amyloid load has usually been observed in the hippocampus [4]. Due to the developmental, structural, and pathological connections between the retina and the CNS and the accessibility of the neuroretina for non-invasive imaging, recent studies have explored retinal Aβ as a potential biomarker for the assessment of cerebral Aβ and AD progression [29,30,31,32,33,34]. Through positron emission tomography (PET) imaging cerebral Aβ accumulation may be detected as early as 20 years before cognitive decline [30,35]. In a meta-analysis using PET imaging, Aβ accumulation was identified in 10–44% of subjects with normal cognition, compared to 27–71% of patients with mild cognitive impairment (MCI) [36]. In a separate meta-analysis, Aβ accumulations were found in 68–97% of AD patients [37]. While PET imaging has been successful in characterizing cerebral Aβ plaques, it is inaccessible for much of the population, costly, invasive, and not suitable for population-based screening for AD risk [30,31]. This warrants further characterization of Aβ in the retinas of AD patients to potentially allow early diagnosis and intervention of AD, which can be crucial for slowing the process of neurodegeneration and preserving cognitive function [38].

### 3.1. Aβ Presentation in the Retinal Enface View and Intra-Retinal Layer View for AD

**Figure 1:**
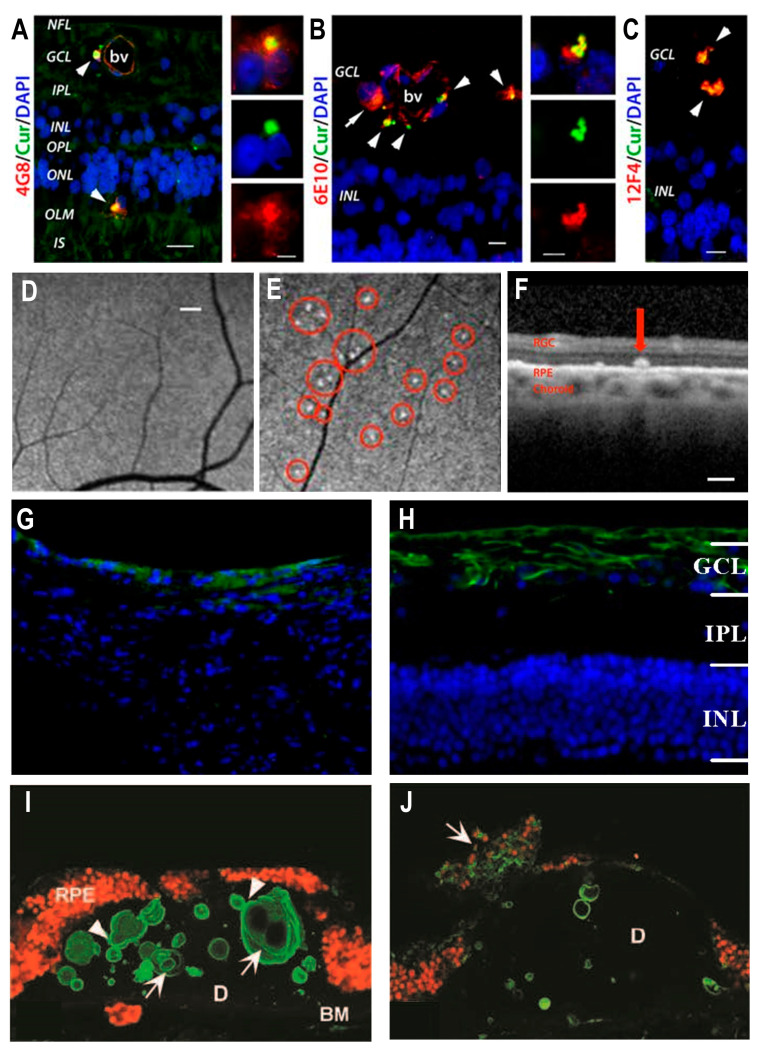
Amyloid-β (Aβ) accumulations in the retina for Alzheimer’s disease (AD), glaucoma, and age-related macular degeneration (AMD). (**A**–**F**) Aβ deposits in relation to AD patients (Cited and modified from [30], used under an open-access license agreement). (**A**–**C**) In retinal donor tissue from AD patients, Aβ accumulations are identified through anti-Aβ monoclonal antibodies (4G8, 6E10, 12F4) and curcumin in multiple retinal layers and especially in the ganglion cell layer. In vivo imaging with curcumin fluorescent probe for (**D**) healthy subject and (**E**) AD patient with Aβ accumulations circled in red. (**F**) Curcumin-positive deposit identified above retinal pigment epithelium (RPE) for AD patients in vivo. (**G**,**H**) Aβ deposits in primate glaucoma model (Cited and modified from [68] used under an open-access license agreement). Anti-Aβ immunostaining show Aβ deposits (green) in (**G**) optic nerve and (**H**) retinal layers, predominantly in the ganglion cell layer. (**I**,**J**) Aβ deposits in AMD retinal donor tissue (Cited and modified from [102] used under an open-access license agreement). Aβ accumulations identified through anti-Aβ monoclonal antibody (green) in (**I**) amyloid vesicles inside drusen located between the RPE and Bruch’s membrane and (**J**) in the RPE.

For post-mortem studies with wholemount retinas and curcumin-based in-vivo imaging studies, increased retinal Aβ was detected in AD patients in comparison to age-matched normal controls (Figure 1). Various Aβ structures were identified including fibrils, protofibrils, and potentially oligomers through transmission electron microscopy of retinal tissue [1,30,39]. Aβ accumulations included both intracellular cyto-deposits and extracellular deposits described by different studies to range from under 5 µm to over 20 µm with larger Aβ deposits resembling a more classical cerebral Aβ plaque [30,39]. Across the plane of the retinal tissue, more Aβ accumulations were identified in the far-peripheral and mid-peripheral of the superior temporal quadrant than in the central retina [30,39,40,41]. Aβ deposits in the superior quadrant were associated with cerebral Aβ deposits, especially in the primary visual cortex (V1) [30]. While the mechanism behind a more superior focused accumulation is currently unknown, this phenomenon may explain the thinning that has been observed in the superior quadrant of the retinal nerve fiber layer, ganglion cell layer (GCL), and inner plexiform layer (IPL), which is made up of the RGC cell bodies and axons [42,43]. Interestingly, early AD patients have predominantly inferior loss of the visual field, which corresponds to the involvement of the superior quadrant of the retina [42,44]. Furthermore, the peripheral loss of RGCs with visual field loss has a similar pattern as what is observed in glaucoma [40].

**Figure 2:**
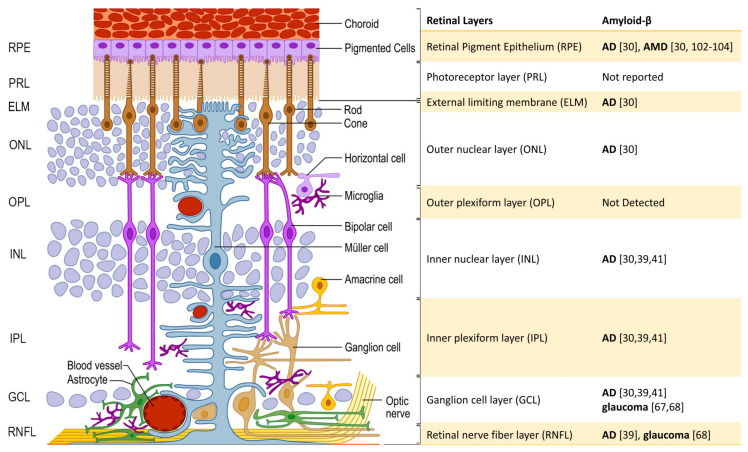
Amyloid-β (Aβ) accumulation in the retinal layers for Alzheimer’s disease (AD), glaucoma, and age-related macular degeneration (AMD) in human and primate studies. Structurally, the neuroretina is made up of distinct layers. The inner retina is composed of the inner limiting membrane (not labeled), retinal nerve fiber layer (RNFL), ganglion cell layer (GCL), inner plexiform layer (IPL), inner nuclear layer (INL), outer plexiform layer (OPL), outer nuclear layer (ONL), and external limiting membrane (ELM). The outer retina contains the photoreceptor layer (PRL) and retinal pigment epithelium (RPE). The retina has distinct alternating layers of synapses (e.g., IPL and OPL) and cell bodies (e.g., GCL, INL, ONL) of neurons (blue) and glial cells [25]. Aβ accumulations are found broadly throughout the retinal layers including within and around neurons and their supporting cells for AD [30,39,41]. For glaucoma, Aβ elevations are more concentrated in the GCL, which includes the beginnings of the optic nerve that are composed of axons of the retinal ganglion cells [67,68]. AMD-associated Aβ deposits are mostly reported within and around the RPE, which allows recycling and maintenance of photoreceptor cells (rods and cones) [30,102,103,104]. (Cited and modified from [120], used under an open-access license agreement).

At the various intra-retinal layers, post mortem human studies showed Aβ deposits above the RPE and located in the inner retina associated with neuronal loss especially within the GCL, inner nucleus layer (INL), and IPL (Figure 1), similar to what was described in AD transgenic mice studies [6,30,45,46]. These findings are in line with Aβ-associated inner retina degeneration and especially RGC dysfunction as detected through decreased pattern electroretinogram responses and positive scotopic threshold response amplitudes found in AD transgenic mice, which may explain the visual disturbances observed in AD patients [1,6,19]. Additionally, increased Aβ-induced toxicity has been suggested to be associated with elevated levels of proteasomal proteins. Decreased levels of proteins associated with synthesis and elongation (e.g., elongation factor EEF1E1) were also observed in the retinas of older AD mice model retinas [47]. In mice models with Aβ positive inner retinas, terminal deoxynucleotidyl transferase dUTP nick end labeling (TUNEL) positive neuronal cells were detected supporting the association between Aβ accumulation and neurodegeneration [1,48]. This association may explain why in vivo human and animal studies show inner retinal layer thinning, especially in the GCL, and loss of ON volume and density [1,18,49]. Intracellular and extracellular Aβ accumulations were the most concentrated in the RGC of the GCL layer (Figure 2) [30,39]. These deposits were also identified inside and surrounding the melanopsin retinal ganglion cells (mRGC), which may be the cause of the mRGC loss and abnormal mRGC morphology in postmortem AD retinas. mRGCs are intrinsically photosensitive and are essential for circadian photoentrainment [40]. In glaucoma, mRGC degeneration was found to induce circadian rhythm dysfunction, which was associated with sleep disturbances [50]. Indeed, mild-moderate AD patients have been associated with reduced sleep efficiency and rest-activity circadian dysfunction, which may be in part associated with the observed Aβ accumulation in mRGCs [40]. Additional research will be necessary to determine if Aβ-induced mRGC degeneration can directly affect sleep efficiency in AD patients or if other underlying mechanisms are involved. Similarly, in both MCI and AD postmortem retinas, Aβ accumulations have been detected in and around pericytes [51], which were found within the endothelial walls of capillaries. Pericytes regulate blood flow and maintain the blood retinal barrier (BRB) and blood brain barrier (BBB) [52]. These accumulations were found to be associated with pericyte loss and possibly contribute to the retinal vascular changes that have been observed in AD patients [51,53].

### 3.2. Relationship between Aβ in the Retina and Brain for AD

Post-mortem and in vivo human studies have also shown an association between retinal Aβ deposits and cerebral structural and functional changes [29,30]. In addition to a positive correlation between supertemporal retinal Aβ42 load and cerebral Aβ, worse neuropathy cortical assessment scores like neuritic plaque score and cerebral amyloid angiopathy (CAA) were correlated to increased retinal Aβ load in AD retinal whole mounts [30,39]. Aβ accumulations positively correlated between ultra-centrifuged homogenates of the hippocampus and the retina [54]. This relationship may explain why retinal Aβ count in the superotemporal retina subregions was inversely correlated with hippocampal volume for both MCI and AD patients in vivo [29]. To note, loss of hippocampal volume has been related to a decrease in executive dysfunction and memory impairment [55,56]. Proximal and mid-periphery retinal Aβ count and surface area were also greater in patients with increased cognitive decline as indicated by low cognitive assessment scores for both MCI and AD patients [29]. These associations support the hypothesis that Aβ-associated neurotoxicity has an associated pathological effect on the retina and brain. However, it is still unclear if accumulated retinal Aβ causes the observed neurodegeneration or if it is simply a downstream product of other disease mechanisms occurring in the CNS. Based on the retinal and cerebral Aβ associations, it may also be possible that neurodegeneration in both tissues may occur simultaneously and mirror each other during AD disease progression.

Based on its accessibility in vivo and correlation with cerebral Aβ levels, retinal Aβ appears to have the potential to become a promising biomarker for early detection of AD-related cerebral changes and cognitive decline. Several techniques have been purposed for in-vivo detection and screening of retinal Aβ including the use of confocal imaging with curcumin, a non-toxic anti-Aβ fluorescent probe, CRANAD-X probes with near-infrared fluorescent imaging (NIRF), and hyperspectral imaging without the need for ingestion or injection of fluorescent probes [5,29,30,31,32,33,57,58,59]. All methods have been able to identify retinal Aβ in vivo and distinguish AD transgenic mice from wild-type (WT) mice [29,30,31,33]. So far, only confocal imaging with orally ingested curcumin has been tested in humans and was able to distinguish AD patients from controls in addition to establishing correlations between retinal Aβ and cerebral manifestations [29,30]. However, since the largest human sample size was 34 patients, further validation in larger sample sizes will be needed to determine if this method can be applied in population-based screening applications [29]. Additionally, repeatability and longitudinal studies are needed to determine if these observed retinal Aβ deposits can be consistently observed and if they persist or change during the AD disease course. While some differences in the presentation of the retinal Aβ can be found between AD and other diseases like glaucoma and AMD, there is still overlapping between the localizations of Aβ (Figure 2). Future studies will also be needed to better characterize retinal Aβ distribution, manifestation, and population prevalence in AD patients, especially in comparison to other amyloidogenic and age-related retinal diseases like glaucoma and AMD that share similar Aβ-associated manifestations in the retina [1,9,60].

## 4. Glaucoma

As one of the top three leading causes of irreversible blindness worldwide [61,62], glaucoma is a multifactorial neurodegenerative disorder that leads to peripheral vision loss [63]. Glaucoma is characterized by RGC degeneration and ON damage that leads to progressive visual field loss, usually associated with acutely or chronically elevated IOP. While lowering IOP has been the mainstay treatment for glaucoma, some patients with glaucoma still experience vision loss even with no incidence of elevated IOP or controlled IOP [64]. This indicates that other factors may be involved in disease progression that works separately or in tangent to IOP-elevation-induced neurodegeneration. Since Aβ is known to induce neuronal apoptosis with associated RGC and visual field loss in AD patients similar to the hallmarks of glaucoma, further exploration of Aβ pathology in glaucomatous eyes is warranted to determine if Aβ plays a role in glaucoma disease progression [65].

### 4.1. Aβ Presentation Associated with Upstream Events in Glaucoma Progression

Aβ accumulation has been identified in post-mortem studies in the retina, especially the GCL and ON in glaucoma patients and animal models (Figure 1 and Figure 2) [1,66,67,68]. In murine models of glaucoma, Aβ was identified in TUNEL-positive RGC from both in vivo and in vitro studies indicating that Aβ directly induces an increase in caspase-3 expression leading to RGC apoptosis [69,70,71]. In WT rat eyes, injected Aβ appeared to have a time and dose-dependent effect on neurodegeneration with increased axonal swelling and increased TUNEL expression in the RGC with GCL thinning and ON injury [71]. Retinal Aβ accumulation may be considered to be one of the upstream events in glaucoma progression [7]. Aβ-associated retinal neuronal cell loss and shrinkage have been associated with astrogliosis and glial activation in the retina, ON, and superior colliculus, where the RGC axons synapse in murine models [7,63,72]. These associations may implicate Aβ in the activation of neuroinflammatory pathways that may lead to progressive glaucoma progression with or without IOP-elevation-related triggers. Furthermore, an increase in Aβ levels was observed to be one of the earliest changes in mouse models of glaucoma in association with the axonal transport deficits of the RGC [2]. Such deficits like cleavage and modification of cytoskeletal proteins have been shown to occur prior to the structural loss and neurodegeneration in glaucoma [73,74]. Due to these upstream associations and its colocalization with degenerative neurons, Aβ has been suggested to cause RGC and other retinal neurons to be more susceptible to stressors like elevated IOP and cause more adverse pathological responses to such insults later in life, eventually leading to a poor disease prognosis [2].

### 4.2. Aβ Presentation in the Retina and Brain in Glaucoma

Initial Aβ accumulation in the retina has been purposed to occur due to stasis of the ocular glymphatic system [75,76]. This system has been identified in the eyes of murine models and appears to be an aquaporin-4 (AQP4)-dependent clearance route found throughout the retina like its cerebral counterpart, which acts as a quasi-lymphatics system for the brain for clearing metabolic waste, including Aβ accumulations [60]. Traced Aβ left the eye through the optic nerve, indicating a glymphatic outflow clearance of Aβ. Furthermore, the outflow was impaired in chronic ocular hypertension and glaucomatous murine models [60]. Thus, stasis of ocular fluid from the retinal neurons has been predicted to lead to pathological Aβ accumulation. The glymphatic outflow is affected by the pressure barrier across the lamina cribrosa where the RGC axons leave the eye to eventually synapse at the brain. An increase in IOP or decrease in intracranial pressure (ICP) can increase this translaminar pressure difference, which may disrupt glymphatic outflow. In addition to elevated IOP being associated with glaucoma, decreased ICP has been reported in primary open-angle glaucoma and in normal-tension glaucoma patients who do not have elevated IOP [75,77].

In Vivo, glaucoma patients had decreased Aβ levels in the vitreous, which corresponds with increased retinal Aβ deposition [7,78]. Interestingly, lower levels of vitreous Aβ were found to associate with lower cognitive function, like in AD pathology [79]. Nevertheless, unlike in AD pathophysiology where cerebral Aβ is most often primarily found in the hippocampus, no Aβ was found in the hippocampus of chronic glaucoma monkey models [7,54]. Instead, Aβ expression was localized to the retina and central visual system, including in the lateral geniculate nucleus (LGN) and weakly in the V1 [7]. Similarly, in glaucoma mouse models, Aβ first accumulated in the retina and then progressed to the ON and later the superior colliculus [14]. For rat models with elevated IOP associated with impaired learning and memory, chronic high IOP was associated with increased Aβ expression in the hippocampus [14]. Therefore, glaucomatous neurodegeneration due to elevated IOP in tandem with Aβ accumulations may originate at the RGC and then progress through the axons forming the ON. This leads to elevated Aβ in the LGN, followed by the V1 of the visual pathway, eventually inducing more AD-like pathology in the brain [7,14,54].

## 5. Age-Related Macular Degeneration

AMD is the most common cause of irreversible blindness in developed countries and especially in people over 60 years of age [80]. Early AMD is characterized by RPE dysfunction with the formation of drusen deposits between the RPE and Bruch’s membrane, generally in the macula, the central region of the retina [81,82,83]. Advanced AMD presents with progressive RPE degeneration and can develop into the exudative AMD (eAMD) with choroidal neovascularization (CNV) or non-exudative AMD (neAMD) with geographic atrophy (GA) [8,84]. While there is no cure for AMD, several vasodilation endothelial growth factor (VEGF) inhibitor therapies have been shown to be safe and effective for long-term prevention of vision loss in eAMD through the indefinite and recurrent treatment of CNV growth and exudation [84,85,86]. However, for neAMD, no such therapy is currently available for mitigating vision loss due to the loss of the RPE, PRC, and inner retinal layers in the regions of GA growth [87]. Since Aβ accumulation has been associated with all stages of AMD progression including drusen, CNV, and GA, recent attempts at identifying new therapeutic targets and interventions have looked toward further characterizing retinal Aβ in AMD [87,88,89,90,91,92].

### 5.1. Aβ in RPE Degeneration and Associated Neuronal Loss

Initial Aβ accumulation may be due to dysregulation of the autophagic system in the retina, especially in the RPE [93]. Autophagy is critical for cellular homeostasis in clearing out unwanted structures through lysosomes [94]. In the retina, the RPE is essential for recycling the PRC outer segments through phagocytosis and other homeostatic functions. Similar to how autophagy has been observed to actively suppress Aβ accumulations in the brain, retinal autophagy appeared to be elevated in human and mice RPE cell lines after being treated with Aβ [93,95]. Detectable RPE activation of autophagy colocalized with Aβ deposits especially in aged human RPE cells [93,96]. Clinical tissue specimens from AMD patients suggested that autophagy is upregulated and impaired in the RPE cells [96,97]. In aged familial AD mice models, which show AMD pathology [98], Aβ was identified in late endosomes and lysosome compartments of retinal neurons, which may destabilize the lysosome membrane and lead to neurotoxic Aβ deposits in the cytosolic compartment [99]. Thus, impaired autophagy may be implicated for Aβ-related neurodegeneration through the RPE cells or directly in retinal neurons.

Aβ accumulation has also been connected to well-established AMD risk factors including high cholesterol and high-fat diets [83,100,101]. In cognitively normal AMD patients, curcumin-based imaging identified Aβ lesions concentrated in the macula and posterior pole. This finding is in line with the general localization of AMD maculopathy (Figure 2) [30]. Additionally, these Aβ lesions were usually larger, more diffuse, and less peripherally located than typical Aβ accumulations found in AD patients [30]. Similarly, in post-mortem studies of AMD retinas, some classical sub-RPE soft drusen were shown to be immunopositive for Aβ [102], which were found in amyloid vesicles that appeared to make up a portion of drusen in donor eyes (Figure 1). These drusen were associated with a disrupted RPE and are often also found in the posterior pole [102,103,104]. Since the RPE functions as a BRB for the neuroretina and plays a key role in the maintenance of the PRC, RPE dysfunction has been considered to the main culprit in AMD progression [105]. Interestingly, in AD mice models, which show AMD pathology, Aβ deposits had been identified inside and around the RPE [106,107]. Increased RPE apoptosis with positive TUNEL staining and caspase-3 expression was observed in human RPE cell lines treated with Aβ [8].

### 5.2. Aβ Presentation in Different Stages of AMD

Even in early AMD, Aβ deposition is considered to potentially sustain chronic inflammation through accumulation throughout the RPE and in drusen [108]. Injection of Aβ in both WT murine models and human RPE cells has also triggered a neuroinflammatory environment with upregulation of inflammasome activation, potentially leading to the death of RPE cells through pyroptosis [8,109]. In AD mice models, Aβ deposits at the RPE have been associated with increased disorganization of the tight junction cells and infiltration of pro-inflammatory-activated microglial cells. Both pathological events can contribute to RPE degeneration [95]. Using a familial AD mice model to study AMD pathology, Aβ accumulations in sub-RPE drusen were observed with early PRC functional deficiencies without PRC cell loss, indicating that Aβ pathology may lead to RPE dysfunction that disrupts its neuroprotective effect for the PRC [91]. Aβ-treated PRC cell lines also showed a dose-response decrease in PRC viability, indicating a direct effect on visual sensitivity in AMD [110]. Since retinal Aβ accumulations in AMD associated drusen prior to neurodegeneration of the PRC, it supports the use of preventative strategies like the use of nutritional supplements and maintaining a healthy diet in early AD [111].

Since increased RPE dysfunction and drusen load has been associated with increased AMD disease severity, Aβ accumulation in the RPE may play a role in progression to advanced AD [106,112]. For eAMD, in human RPE cell lines, high doses of Aβ-induced RPE apoptosis and increased expression of VEGF. As a signaling protein that promotes vessel growth, VEGF in the retina can lead to increased angiogenic cytokine levels and susceptibility to abnormal vessel growth in the macula, potentially progressing to eAMD with CNV [8,113,114]. Low concentrations of Aβ were associated with an increased level of pigment epithelium-derived factor, which had a neuroprotective effect and inhibited apoptosis of RPE cells, thus leading to an increased RPE proliferation and cell count [113]. Therefore, it appears that increased amounts of Aβ can overwhelm protective mechanisms in the RPE against stressors.

For neAMD, while the exact mechanism behind GA is unknown, growth of GA has been associated with deposition of inflammatory cellular debris in the Bruch’s membrane leading to the hyperactivation of the alternative complement pathway and resulting in RPE cell death [87,115,116]. Thus, Aβ deposition may be the main culprit behind GA pathology. In spheroid cultures of human RPE cells, “stressed” RPE spheroids produced an increased amount of drusen, which contained Aβ and complement factors [81]. Similarly, in transgenic mice that overproduce Aβ, increased drusen-like deposits with Aβ and complement proteins were identified with increased glial cell response [117]. Furthermore, Aβ has been shown to inhibit complement factor I (CFI). A missense CFI variant is one of the loci associated with AMD [118]. CFI decreases activation of the alternative complement pathway by cleaving C3b to iC3b [119]. Therefore, increased Aβ deposition may lead to the GA-associated hyperactivation of the complement system, which may contribute to retinal neuronal loss through GA progression [116].

## 6. Treatment Approaches for Reducing Aβ Accumulation

Aβ accumulations in the retina have been suggested to be an overlapping aspect in the pathophysiology of disorders like AD, glaucoma, and AMD, which do not have a cure and can contribute to irreversible neurodegeneration [121]. Recent clinical trials have tested treatment approaches that focused on reducing Aβ deposits in the retina [4,116,122,123,124,125,126,127]. The mechanisms of action mostly included direct binding and clearance of Aβ through monoclonal (mAb) anti-Aβ antibodies, use of β-secretase inhibitors to reduce the production of Aβ, or use of polyclonal amyloid antibodies that also decreased Aβ accumulations [4,116,122,123,124,125,126,127]. While many treatment approaches were able to generally decrease Aβ load [127,128], most did not appear to be able to achieve the primary objective of mitigating disease pathology.

### 6.1. Anti-Aβ Antibody-Associated Clinical Trials for AD

In patients with AD, multiple mAb anti-Aβ systemic intravenous treatments (i.e., aducanumab, gantenerumab, and solanezumab) were applied and showed promise in removing Aβ through systematic dosing by increasing clearance through binding, preventing aggregation, or through Fc-mediated phagocytosis in MCI and AD patients [129,130,131]. However, no evidence of cognitive improvement was seen in the treatment group for mAb anti-Aβ antibodies in comparison to the placebo group in phase III trials for prodromal, mild, and mild-to-moderate AD [122,123,124]. Similarly, beta-site APP cleaving enzyme 1 (BACE1) inhibitors (i.e., verubescestat and CNP520), which decrease the rate of proteolytic cleavage of APP to Aβ, also showed promise in reducing Aβ load, but were unsuccessful in reducing cognitive decline or preventing future cognitive decline in cognitively normal older adults and AD patients [125,126,132]. It has been suggested that a longer observation period or higher level of dosing may have been needed to see a difference between groups since AD-associated cognitive decline may occur over decades especially in prodromal AD. However, higher doses may lead to increased occurrence of side effects like microhemorrhages that have been observed during clinical trials [124,133]. Additionally, no protective effects of cognition were observed in patients with more severe AD that has a more rapid rate of cognitive loss [123,124,125]. This indicates that the Aβ-disaggregating effects of anti-Aβ fluorescent probes like curcumin or near-infrared imaging probes, as shown in the brain and retina of AD mice models, may not have much potential as therapeutic agents [32,58]. Furthermore, the suppressive effect of curcumin on Aβ accumulation appears to weaken the correlation between retinal Aβ and cerebral Aβ levels, which may affect its ability to be used in early detection and tracking of AD onset and progression in vivo through retinal imaging [58].

On the other hand, some limited success has been observed in intravenous immunoglobulin (IVIG) treatment [127]. IVIG contains polyclonal variants of naturally occurring antibodies against Aβ, which led to increased binding and clearance of Aβ. IVIG demonstrated the ability to reduce Aβ in both the retina and the brain after 2 months of therapy for patients with MCI in a proof of mechanism study [4]. Yet like mAb antibodies, no cognitive benefits were seen in a phase III trial for mild to moderate AD patients [134]. IVIG did show promise in preventing cognitive loss in MCI and prodromal AD. In a retrospective study, previous treatment for IVIG showed a lower risk of developing AD-associated dementia after 5 years for a predementia MCI group [135]. Furthermore, the IVIG-treated MCI group showed increased preservation of cognitive function with less brain atrophy. The treated group also showed decreased conversion to AD after 1 year of study in comparison to the placebo group in a Phase II trial. However, no difference in cognitive difference was observed between groups after two years [127]. These results may indicate that IVIG has a limited protective effect on cognitive decline that becomes less significant after 1 year. Nevertheless, it is unknown if the neuroprotective effect of IVIG arises from its anti- Aβ components or from the combination of its more varied mechanisms of action that allows IVIG to also attenuate inflammation and bind other neurotoxic peptides like hyperphosphorylated tau [136,137]. Therefore, this may indicate that a more multifactorial treatment may be beneficial for treating AD pathology, which will need to be validated in future studies and clinical trials.

### 6.2. Aβ-Associated Clinical Trial Targets for Glaucoma and AMD

For the neurodegeneration-associated ocular disorders, some treatment approaches for reducing Aβ accumulation have been explored. In glaucoma, when given systemically in murine models, α2 adrenergic agonists (α2ARA) like brimonidine (BMD) show promise in reducing Aβ production by promoting the non-amyloidogenic pathway, which elevates sAPPα levels [24]. sAPPα appeared to act as a BACE1 inhibitor for transgenic APP mice in vivo [138]. Use of this α2 agonist in an ocular hypertensive glaucoma rat model showed decreased Aβ, which was associated with decreased RGC apoptosis and axonal degeneration in comparison to that of a non-treated rat [24]. BMD’s IOP-independent neuroprotective effects may also explain why low-pressure glaucoma patients appeared to have less visual field loss after the use of BMD eyedrops, if they did not develop an ocular allergy [139]. BMD was shown to be safe in humans as an intravitreal implant in a phase II trial, which may indicate that its benefits for treating glaucoma-related amyloidosis may outweigh the risk of its potential adverse effects [128]. Interestingly, BMD has also been shown to have some limited success in slowing GA growth in neAMD [128]. On human RPE cell lines, BMD has been shown to have a similar neuroprotective effect even when the RPE cells were treated with stressors [140,141]. In a phase II trial, an intravitreal implant of BMD slowed GA growth in the treatment group in comparison to the sham group only at Month 3, with no differences between groups for the primary study endpoint at Month 12 [128]. This may indicate that the protective effect wanes after the initial beneficial reaction and a higher or more frequent dosage will be needed to sustain the effect on GA growth.

Similarly, a mAb anti-Aβ was also tested in GA secondary to neAMD [128]. mAb anti-Aβ antibodies associated with Fc-mediated phagocytosis of Aβ were predicted to reverse the Aβ inhibition of CFI. Increasing activation of CFI decreases C3b levels, which should decrease alternative complement activation [108,116]. In AMD-associated murine models, mAb anti-Aβ was shown to reduce Aβ accumulation and complement activation that has been purposed to lead to GA growth [92,142]. Nevertheless, no difference in GA growth was detected for the intravenous mAb anti-Aβ antibody-treated group in comparison to that of the placebo group in a phase II clinical trial for human mAb GSK933776 [116]. Since the mAb antibody was given intravenously, perhaps not enough antibody accumulated at the RPE and other areas of maculopathy in the retina. Alternatively, similar to AD treatment, therapeutic interventions for GA may be needed to be given at an earlier stage of AMD to be effective for slowing the growth of GA. Treatment prior to GA onset may be more effective for preventing unrecoverable RPE degeneration and PRC loss. Since GA progression has been demonstrated to involve multiple underlying mechanisms, a multifactorial treatment that can target Aβ and its associated pathological cascades may prove to be beneficial [112].

### 6.3. Conclusions for Current Aβ-Associated Interventional Targets and Future Directions

**Figure 3:**
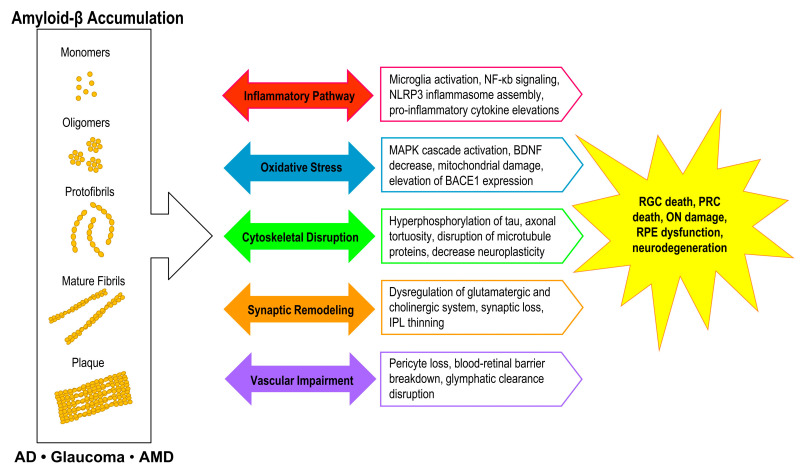
Flowchart for overlapping retinal amyloid-β (Aβ)-associated mechanisms and effects in AD, glaucoma, and AMD. Elevated Aβ levels in various structural formations, especially oligomers and plaques, have been associated with increased upregulation of pathology cascades. These pathways have been associated with increased Aβ accumulations and increased neurodegeneration with a potential loss of cognition and vision. Abbreviations: AD: Alzheimer’s disease; AMD: age-related macular degeneration; NF-κb: nuclear factor kappa B; NLRP3: NLR family pyrin domain containing 3; MAPK: mitogen-activated protein kinase; IPL: inner plexiform layer; RGC: retinal ganglion cell; PRC: photoreceptor cell; ON: optic nerve: RPE: retinal pigment epithelium.

Ultimately, while many treatment trials appear to be generally successful in removing or preventing Aβ accumulations, most treatments have not been as successful in mitigating cognitive decline or vision loss in these disorders. While this may be a result of insufficient Aβ removal or due timing of the treatment in the disease course, it may also indicate that the treatment may have been too specific to removing Aβ without treating the existing pathological cascades that had already been triggered or amplified by the presence of Aβ deposits. Several Aβ-associated effects appear to be overlapping between AD, glaucoma, and AMD including an increase in the inflammatory pathways, oxidative stress, cytoskeletal disruption, synaptic remodeling, and vascular alterations (Figure 3). Further understanding of these mechanisms may provide new targets for potential therapeutic interventions against neurodegenerative diseases that may yield more optimistic outcomes.

### 6.4. Inflammatory Pathway

Aβ accumulation in the retina has been demonstrated to upregulate inflammatory pathways, which has been associated with increased retinal neurodegeneration (Figure 3) [143]. Upregulation of microRNA that target genes regulating pathways linked to neurodegeneration and inflammatory cascades was observed in relation to elevated Aβ levels in vivo leading to a pro-inflammatory environment with retinal cell loss and break down [143,144]. In AD, glaucoma, and AMD, colocalization of Aβ deposits and microglia indicated with iba-1 positive cells has been identified in transgenic mice models [8,66,72,107,145]. Activated microglia were shown to displace toward more vertical formations that may distribute signaling to different microglia plexuses throughout the retinal layers including the IPL, INL, and RPE, which can then migrate and surround Aβ deposits [45,48,117,145,146]. While acute activation appeared to decrease Aβ accumulation through phagocytosis, chronic activation of microglia led to a more proinflammatory milieu that was linked to increased neurotoxicity [45]. This may be associated with the presence of the CD36 receptor on microglia that form Aβ-CD36 complexes, which elicits phagocytosis but also induce pro-inflammatory cytokine release. In CD36 knockout mice, injection of Aβ did not trigger the inflammatory cascade that was associated with RGC death [72].

#### 6.4.1. NF-κb Signaling Pathway and Associated Inflammatory Cascades

Aβ-inducted microglia activation was found to associate with upregulation of the nuclear factor kappa B (NF-κb) inflammatory pathway with enhanced expression of pro-inflammatory elements. In Aβ-treated human RPE cells, increased RelA, RelB, and c-Rel signaling of the NF-κb family, all appeared to cause pro-inflammatory cytokine and RPE degeneration with silencing of the transcription factors leading to decreased cytokine release [147]. Aβ has been shown to lead to enhanced levels of the NF-κb inflammatory pathway elements like interleukin 6 (IL-6), IL-8, tumor necrosis factor α (TNFα), p16, and matrix metallopeptidase 9 (MMP-9) that contributed to RPE degeneration and PRC death in murine models and human RPE cells [90,91,95,119,148]. Thus, the NF-κb inflammatory pathway may cause PRC loss in neurodegenerative diseases, especially in AMD where RPE degeneration is closely linked to PRC loss. When pro-inflammatory cytokines like IL-6 and TNFα were reduced through the treatment of transforming growth factor β1 in WT rats injected with Aβ, decreased apoptotic markers were detected in the rat neuroretina [149]. In particular, MMP-9 has been shown to impair the barrier integrity of RPE cells through chronic inflammation with proteolysis of tight junction proteins such as zonula occludens-1 and occludin, which leads to increased RPE cell senescence [95]. Inhibition of NF-κb signaling through an IkB kinase inhibitor also prevented the disruption of RPE tight junctions in Aβ-treated WT mice [106]. Furthermore, when WT mice were treated with elovanoids, a lipid mediator, which has been shown to downregulate the gene expression of Aβ-associated inflammatory components, the RPE and PRC of Aβ-injected mice retinas were spared from degeneration [91]. This supports the direct effect of Aβ neurotoxicity on the PRC and indicates that inhibition of several elements in this signaling pathway may be beneficial for preventing neuronal loss.

Increased Aβ neurotoxicity and increased NF-κb signaling have also been associated with NLR family pyrin domain containing 3 (NLRP3) inflammasome assembly and activation [48,89]. NLRP3 inflammasome activates caspase-1 and causes the maturation of interleukin 1 beta (IL-1β), a proinflammatory mediator that may also activate IL-6, and IL-18, which is associated with interferon-gamma production [150]. The components of this pathway lead to pyroptosis proinflammatory cell death, which has been observed in WT mice injected with Aβ and human RPE cells treated with Aβ [8,89,151]. In AD, a higher expression of IL-1β was detected in microglia of postmortem AD retinas in comparison to controls, which indicated inflammasome activation and is in line with the expression of microglia in the brains of AD patients [48,152]. Interestingly, overexpression of angiotensin-converting enzyme 2 prevents upregulation of IL-1β, decreased leukocyte recruitment, and cytokine release in Aβ-treated human RPE cells, which indicates that an axis of the renin-angiotensin hormonal system has a protective effect on retinal cells against Aβ-associated inflammation [151]. Furthermore, upregulation of liver x receptor α also protected against Aβ-associated inflammasome activation in RPE cells in vivo [89,90]. Future research may determine if inhibition of these Aβ-induced inflammatory pathways will mitigate neurodegeneration, especially in AMD where RPE dysfunction plays a major role in disease progression.

#### 6.4.2. Complement System Activation

Furthermore, Aβ accumulation has also been associated with activation of the complement system leading to the formation of membrane attack complexes and increased inflammation-inducing retinal neurodegeneration [48,108,119]. Evidence of both the classical and alternative complement system has been identified in Aβ-injected WT mice retinas with the increased expression of multiple complement factors [119]. Complement C3 was upregulated in astrocytes of postmortem retinal tissue, which indicates that activation of the complement cascade in AD retinas may be associated with neurodegeneration [48]. As previously described, Aβ accumulation has been purposed to lead to increased alternative complement activation through inhibition of CFI, which increases C3 production and may contribute to increased GA progression in neAMD [108]. While direct inhibition of Aβ has so far been unsuccessful in decreasing GA progression [116], direct local C3 inhibition did reduce GA growth rate in comparison to a sham group in a phase II trial [153]. Altogether, several potential therapeutic measures may be associated with the inflammatory pathways associated with retinal Aβ accumulation. Direct targeting of these elements may yield positive results for neurodegeneration-associated diseases that affect the retina.

### 6.5. Oxidative Stress

Since the retina is one of the most energy-demanding and oxygen-consuming tissues in the human body, the retinal neurons are highly susceptible to Aβ-associated oxidative stress, which may contribute to neurodegeneration in AD, glaucoma, and AMD (Figure 3) [95,154,155]. Aβ accumulation in the retina increases oxidative stress markers in a time and dose-dependent manner, which may induce the observed increased RGC apoptosis, ON damage, glial cell activation, and RPE degeneration [109,156]. Aβ-induced oxidative stress with increased production of reactive oxygen species (ROS) may act as a second messenger to activate the mitogen-activated protein kinase (MAPK) cascade, which can upregulate the NF-κb inflammatory pathway and assembly of NLRP3 inflammasomes leading to increased neurodegeneration [109,155,157]. Inhibition of c-Jun N-terminal kinase signal pathway, which is part of the MAPK superfamily, reduced neurodegeneration in a transgenic APP mouse model with increased Aβ accumulation [155]. Aβ accumulation also appeared to decrease the levels of the brain derived neurotrophic factor, a neuroprotective neurotrophin, which when restored lead to decreased oxidative markers and appeared to prevent Aβ-induced RGC apoptosis and ON damage in Aβ-injected WT rats [71,156]. Therefore, prevention of Aβ-associated oxidative stress may provide potential treatments for mediating neurodegeneration, especially for glaucoma where RGC and ON are the most directly affected.

Aβ-associated oxidative stress may also be due to DNA alteration. Since the retina has high metabolic damage, mitochondria function is crucial for cell maintenance [158]. Mitochondrial DNA (mitoDNA) is susceptible to Aβ-induced oxidative damage due to poor DNA repair capacity, which can induce increased cell stress and eventually cause cell death [159]. In cybrid human RPE cells treated with Aβ that contained mitoDNA from AMD patients, an upregulation of ROS species was observed with increased apoptotic markers and decreased cell viability in comparison to treated cybrid cells with mitoDNA from normal controls [3]. The oxidative effects of Aβ in AMD cybrids were reversed by treatment of mitochondria-derived peptides, which are documented to have a protective effect against Aβ-associated oxidative stress and noted for its effect against AD [3,160]. Furthermore, increased accumulation of Aβ may be due to oxidative stress-induced DNA methylation alterations. In photo-oxidative damaged human RPE cells, decreased levels of DNA methyltransferase 1 were observed with abnormal methylation of specificity protein 1 (SP1) binding sites in the BACE1 promoter, which appeared to increase BACE 1 production through increased binding of SP1 to the BACE1 promoter. Inhibition of SP1 through mithramycin A treatment led to the downregulation of BACE1 and decreased Aβ accumulation and oxidative stress-associated RPE degeneration [27]. In particular, AMD includes risk factors such as chronic photo-oxidative light exposure and smoking, which can cause oxidative stress in the retina. This in turn may lead to increased Aβ accumulation in the retina [27,161,162]. Indeed, AMD patients have also been shown to have impairment in DNA methylation [163]. Future studies are needed to determine if prevention of the pathway components associated with Aβ-induced oxidative stress can prevent irreversible neurodegeneration and decrease disease progression.

### 6.6. Cytoskeletal Disruption

Cytoskeletal protein and organization modifications in retinal neurons have been observed in relation to Aβ accumulation, which has been implicated in AD, glaucoma, and AMD (Figure 3) [110]. Aβ neurotoxicity may be due to its potential to affect the assembly and disassembly of cytoskeleton proteins and lead to decreased cell viability [164]. Cytoskeletal modifications appear to occur prior to more widespread neurodegeneration [2]. Loss of retinal neurons like the PRC has been observed in AD, glaucoma, and AMD [110,160]. In a WT mouse PRC cell line, Aβ treatment led to increased actin cytoskeletal reorganization and decreased cell viability in a dose and time-dependent manner [110]. Aβ treatment also appeared to induce phosphorylation of tau, a microtubule-associated protein, which has been found in neurofibrillary tangles and cytoskeletal pathology in AD [110,165]. When Aβ-injected transgenic tauopathy mice models were treated with Epothilone D, a microtubule stabilizer, the increased Aβ-associated tau hyperphosphorylation and aggregation was prevented, which reduced RGC and axonal loss [166]. Therefore, this supports existing studies and potential treatments that aim to directly treat tauopathy for AD.

Similarly, WT rats injected with Aβ showed a downregulation of retinal neuronal microtubule-associated protein 2 (MAP-2), which is involved in the microtubule of axons and dendrites in addition to affecting the neuroplasticity of post-mitotic neurons [83]. MAP-2 impairment has been observed in advanced AMD with MAP-2 identified in abnormal PRC with increased axonal tortuosity and irregularly located nuclei of postmortem retinal tissue with GA [167,168]. Therefore, Aβ accumulations may be related to AMD phenotypes that show PRC loss prior to RPE degeneration. Aβ-treated RPE cells also showed actin cytoskeleton disorganization, which further decreases PRC cell viability [169]. Early cytoskeletal modifications have also been observed in a transgenic glaucoma mouse model with Aβ elevations. Increased Aβ accumulation has been purposed to increase intracellular calcium concentration, which can modulate calpain, a calcium-dependent protease that can cleave structural proteins of the cytoskeleton [170]. Increased calpain has been observed in the same mice model even without elevations of IOP. Therefore, Aβ-associated early cytoskeletal disruptions may warrant further studies to identify elements that may be modified to prevent further disease progression.

### 6.7. Synaptic Remodeling

Aβ accumulations were found to be associated with synaptic cleft remodeling. In AD, glaucoma, and AMD, increased Aβ elevation appears to cause dysregulation of neurotransmitter levels, which has been implicated in increased excitotoxicity and synaptic loss (Figure 3) [24,146,171]. Murine models of age-related neurodegeneration show Aβ deposits in the retina and dysregulation of glutamate levels at the synaptic cleft with activation of N-methyl-D-aspartate (NMDA) receptor activation leading to increased neuronal degeneration [146,172]. Glutamate is the main neurotransmitter of the retina and glutamatergic synapses are found in the outer plexiform layer and IPL of the retina. Indeed, Aβ deposits have been located in the IPL in postmortem AD retinas in comparison to healthy controls [30]. This may also explain thinning of the IPL that was observed in mild-to-moderate AD patients since these layers contain the synapses of the retinal neurons [65]. Thinning of the IPL has also been observed in glaucoma and AMD patients in vivo, which may suggest an associated synaptic degeneration. Additional studies would be necessary to determine if the thinning is due to Aβ accumulation [173,174]. When aging AD mice models were immunized with glatiramer acetate, the retinal and brain glutamine synthetase levels became comparable to healthy WT mice, which appeared to restore glutamate levels, reduce excitotoxicity, and reduce synaptic loss [175]. Similar effects were observed with high concentration dosing of α2ARA, which appeared to modulate excitotoxicity and decrease excessive glutamate at synaptic clefts, which may help explain its potential effect in mitigating glaucoma and neAMD progression [24,128]. Thus, targeting early synaptic changes in these disorders may prevent progression to Aβ-induced neurodegeneration.

Pre-clinical AD patients have also shown early disruption of the cholinergic system in association with retinal Aβ load [171]. In muscarinic acetylcholine receptor knockout mice as an AD model, decreased activation of cholinergic receptors co-occur with increased Aβ load [176]. Postmortem studies showed decreased acetylcholinesterase and choline acetyltransferase activity in cognitively normal elderly subjects at risk for AD [177]. Similarly, in a cognitively normal population with cerebral Aβ load, increased area of inclusion bodies with Aβ correlated with IPL thickness, especially near the ON [171]. The IPL is a cholinergic-rich retinal layer that contains the retinal synapses that are the closest to the brain [178]. Early changes in the IPL may indicate dysregulation of cholinergic synapses that can lead to synaptic loss with disease onset and progression. Future studies on the effects of synaptic cleft dysfunctions in association with Aβ may help determine if similar changes are observed in early glaucoma and early AMD disease due to IPL alterations and Aβ accumulations also being observed in these aging-related neurodegenerative diseases.

### 6.8. Vascular Alterations

Elevated Aβ has been detected in and around the retinal vasculature in postmortem and in vivo studies, which has been purposed to affect the vessel structure and perfusion [51,179]. Vascular risk factors exist for AD, glaucoma, and AMD, which have been considered to play a role in disease pathogenesis (Figure 3) [29,180,181]. In AD, postmortem retinas showed Aβ deposits within and around isolated pericytes. Increased pericyte loss and decreased vascular platelet-derived growth factor receptor-β (PDGFRβ) signaling were also observed, which is associated with BRB breakdown. This mechanism reflects what has been described in the brain in association with BBB breakdown, which has been demonstrated to be related to cognitive decline in AD patients [51,182,183]. Furthermore, PDGFRβ deficiency was associated with increased retinal vascular Aβ burden and worse neuropathy cortical assessment scores including increased CAA severity, increased brain Aβ plaques, and decreased cognitive status [51]. This indicates that a parallel vascular amyloidosis mechanism may occur in the BBB and BRB in patients with AD, which further supports the use of retinal Aβ as a biomarker for early detection of AD disease progression [184]. For AD patients in vivo, impairment of retinal neurovascular coupling was also inversely correlated with level Aβ in the cerebral spinal fluid, a surrogate of cerebral Aβ [185]. Since both cerebral and retinal blood flow has been observed to be low in AD, the resulting hypoperfusion may impair endothelial function and production of nitric oxide, which may lead to further vessel impairment and Aβ accumulation in vessels [185,186]. Of note, some studies that compared healthy subjects with or without cerebral Aβ load did not observe any retinal vascular differences between groups [53,187]. Therefore, additional underlying mechanisms may contribute to the observed vascular alterations in AD outside of amyloidosis.

Similarly, in retinal neurodegenerative disorders, Aβ accumulation may be associated with changes in the retinal vasculature. In glaucoma, vascular impairment has been observed with dysregulation of vascular pulsation, which may affect glymphatic clearance leading to increased Aβ accumulation, especially at the lamina cribrosa where the ON exits the eye [75,188]. Hemorrhages have also been located in the optic disc [189]. Indeed, elevated Aβ has been observed at the ON head tissues from postmortem glaucomatous eyes, which may help explain these vascular changes [67]. Furthermore, Aβ accumulation was observed in the retina and choroidal vessels of an AMD mice model, which has been shown to influence blood flow dynamics and lead to increased endothelial cell apoptosis, potentially due to increased susceptibility to Aβ-induced inflammation [179]. Dysfunction of retinal and choroidal vessels increases the risk of PRC loss since they supply oxygen and nutrients to the outer retina. Additionally, elevated Aβ levels in human RPE cells showed increased expression of VEGF, which are implicated in the growth of CNV in eAMD [113]. Therefore, new treatment targets that can mitigate Aβ-associated vascular impairment may be beneficial for preventing disease progression in these neurodegenerative disorders.

## 7. Conclusions and Future Directions

Retinal Aβ accumulations in neurodegeneration-associated disorders like AD, glaucoma, and AMD have been extensively studied and regarded as an overlapping pathological feature between these disorders with no successful cure. Found in various retinal layers, Aβ has been characterized extensively in animal and human studies. In AD, retinal Aβ accumulations have been shown to be associated with Aβ load in the brain and correlated with metrics of cognitive functioning. In glaucoma and AMD, Aβ has been identified in the RGC and RPE, respectively, which are directly affected by the disease pathology. Importantly, Aβ accumulation in the retina and associated pathology have been observed to occur prior to irreversible neurodegeneration in these diseases, which indicates its potential to be a biomarker for early detection of disease progression. Due to the connections and parallel pathways between the retina and the CNS and the accessibility of the neuroretina for non-invasive imaging, the retina has provided a unique opportunity for tracking and understanding the alterations in the brain that may occur during neurodegeneration. However, retinal Aβ imaging is currently not ready to be applied for population-based screening. Future studies are needed to further characterize the distribution, manifestation, and prevalence of retinal Aβ among AD, glaucoma, and AMD to better differentiate these amyloidogenic pathologies prior to widespread screening for early detection of AD. Additionally, longitudinal studies will also be necessary to determine if retinal Aβ loads persist over time and how these deposits change with disease progression.

Furthermore, while retinal Aβ has been shown to contribute to increased neuronal loss in line with these pathologies, treatment approaches directly targeting Aβ accumulation systematically and locally has largely been not effective at mitigating disease progression and achieving the primary objective of preserving cognition and vision in various stages of disease severity. This may indicate that either not enough Aβ deposits had been removed to produce a measurable clinical effect or Aβ accumulations had already initiated pathological cascades that have led to progressive neurodegeneration. Thus, Aβ-based treatment for AD, glaucoma, and AMD still requires further development and exploration before it may be beneficial for clinical application. One possible direction for new treatment approaches may involve directly targeting the pathological mechanisms that are associated with retinal Aβ accumulation. Among the many Aβ-associated pathological mechanisms, the pathophysiology of AD, glaucoma, and AMD appear to be affected by Aβ-induced inflammatory pathways, oxidative stress, cytoskeletal disruption, synaptic remodeling, and vascular alterations. Understanding of the mechanisms behind these shared pathological effects has yielded several potential therapeutic targets that may provide possible treatment targets for these neurodegenerative diseases. Future studies based on the pathway components may lead to therapeutic measures that can ameliorate the pathological effects of these neurodegenerative diseases and protect against loss of cognition and vision. Ultimately, for AD, glaucoma, and AMD, Aβ accumulation in the retina with its associated pathological cascades may hold the key for further understanding the pathophysiology of complex neurodegeneration-associated diseases, which can potentially lead to novel treatments and clinical applications.
